# Prevalence and factors associated with severe vitamin D deficiency in HIV/hepatitis C co-infected patients

**DOI:** 10.1186/1758-2652-13-S4-P103

**Published:** 2010-11-08

**Authors:** S Surah, A Samarawickrama, L Campbell, R Kulasegaram, F Post, M Fisher, B Peters, J Fox

**Affiliations:** 1 Guy's & St Thomas' NHS Foundation Trust, Department of GUM & HIV, London, UK; 2 Brighton & Sussex University Hospital, Clinical Investigation & Research Unit, London, UK; 3 King's College Hospital NHS Foundation Trust, Department of HIV/GUM, London, UK; 4 Brighton & Sussex University Hospital, Department of GUM & HIV, London, UK

## Purpose of study

Vitamin D deficiency (VDD) is associated with elevated risks of cardiovascular disease, malignancies and impaired survival of the general population. In HIV infection VDD is related to anaemia, HIV disease progression and death, and in hepatitis C (HCV) infection impaired treatment responses to interferon. Severe VDD is common in both HIV and HCV mono-infection. The prevalence of VDD in HIV/HCV co-infected patients, and the effect of the severity of liver disease on vitamin D status remains unknown. The aim of this study was to investigate the prevalence of, and factors linked to severe VDD in HIV/HCV co-infected patients.

## Methods

Multi-centre observational study of 309 HIV/Hepatitis C co-infected and 128 HIV mono-infected patients matched for gender and ethnicity. Patients included attended between Sept 09 - July 2010. Severe VDD was defined as 25(OH) vitamin D level <25 nmol/L. Database analysis and case note review was performed. Multivariate logistic regression was used in a model incorporating gender, ethnicity, and season of sample to examine associations between severe VDD, parathyroid hormone (PTH) and HCV status. Patients on vitamin D supplementation were excluded from analysis.

## Summary of results

91% of patients were male, 86% were Caucasian and 18% of HCV had been acquired through intravenous drug use. The prevalence of severe VDD in HIV/HCV co-infected and HIV mono-infected patients was 19% and 6% respectively (p=0.876). The median vitamin D concentration was 29 (range 7-135) and 46 (11-168) nmol/L (p=0.396), and the median PTH concentration was 41 (Range 12-241) and 36 (11-197) units respectively. In HIV/HCV patients, severe VDD was associated with winter season (October to March) (p=0.0001), black ethnicity (p=0.0001), and higher fibroscan score (p=0.05), but not with age, HIV or HIV viral load, HCV genotype, ALT, ALP, platelets or HCV treatment status.

## Conclusions

This is the first report on the prevalence of VDD in patients infected with HIV/HCV. Severe VDD deficiency was not associated with HIV/HCV co-infection. In HIV/HCV co-infected patients severe VDD was linked to winter season, black ethnicity and liver fibrosis. Further investigation of the relationship between vitamin D deficiency and liver fibrosis in HIV/HCV co-infection is warranted.

